# The “Swiss-cheese Doppler-guided laser tonsillectomy”: a new safe cribriform approach to intracapsular tonsillectomy

**DOI:** 10.1007/s10103-012-1140-1

**Published:** 2012-08-02

**Authors:** B. Palmieri, T. Iannitti, G. Fistetto, V. Rottigni

**Affiliations:** 1 Department of General Surgery and Surgical Specialties, University of Modena and Reggio Emilia Medical School, Surgical Clinic, Modena, Italy; 2 Department of Physiology, School of Medicine, University of Kentucky Medical Center, Lexington, KY 40536-0298 USA; 3 Poliambulatorio del Secondo Parere, Modena, Italy

**Keywords:** Neodymium-YAG laser, Tonsil, Tonsillectomy, Surgery, Head and neck, Inflammation, Pain

## Abstract

Outpatient laser ablation of palatine tonsils is a very interesting procedure that has been recently introduced as a routine in head and neck surgery departments. The aim of this study was to describe a new strategy using a Doppler-guided fibre optic neodymium-yttrium–aluminium–garnet (YAG) laser to remove up to 80 % of tonsillar tissue, as assessed in the long-term postoperative clinical evaluation of the volume of the tonsils at the follow-up, and leaving the capsule in place, thus avoiding any haemorrhagic complication and minimize pain. A total of 20 patients (men, *n* = 13; women, *n* = 7), aged between 6 and 63, were recruited for the procedure. They were affected by chronic hypertrophic tonsillitis with a recurrent fever and other symptoms that were related to oral inflammation. Among the 20 patients, no serious adverse events, including haemorrhage-related complications, were observed. Treatment was well tolerated, even in patients displaying an overall low pain threshold. No dropout or uncompleted procedure occurred in the present study. Minor complications included sore throat, moderate oedema, mild acute pharynx inflammation, slight peritonsillar exudate and local burning. The postoperative pain, measured by Scott–Huskisson visual analogue scale, was between 5 and 40 mm and was easily counteracted by means of external ice packages and nonsteroidal anti-inflammatory drugs, according to the individual patient’s need. During the 12–36-month follow-up patients showed improved symptoms (*n* = 7) and complete recovery (*n* = 13). A relapse episode was observed in two patients. This study supports fibre optic laser neodymium-YAG tonsil surgery, named “cribriform intracapsular tonsillectomy” or “Swiss-cheese laser tonsillectomy”, as an effective alternative to the traditional cold knife approach or electrosurgery. This approach could become the gold standard for tonsil surgery in the third millennium for safety reasons, acceptable cost–benefit ratio, the precise targeting of the beam across the affected tissues and the short- and long-term recovery.

## Introduction

Outpatient laser ablation of palatine tonsils is a very interesting procedure that has been recently introduced as a routine in head and neck surgery departments [2, 7, 8]. The traditional technique of “cold” dissection was introduced about 100 years ago [12]. Since then, many different techniques have been introduced to speed up the operation, keep intraoperative bleeding to a minimum and reduce postoperative morbidity. In Krespi and Ling’s [7] case series, laser tonsillar tissue vaporization under local anaesthesia is successfully reported in 96 % of patients during a 4-year follow-up period. On the other hand, general anaesthesia is required as a routine procedure for laser tonsillotomy or tonsillectomy, in children affected by chronic inflammation or sleep apnoea. According to some authors [2, 5, 8], during a single or multiple 15-min sessions, it is possible to remove the infected tonsillar crypts, leaving in place the capsule as well as almost 30 % of the deep tonsillar tissue. In adults, palatine tonsillectomy, with capsule excision for chronic recurrent tonsillitis, is also a classic technique requiring general anaesthesia with a risk of complications and postoperative pain. Andrews and Latif [1] described a local anaesthesia modification of Krespi and Ling’s [7] technique using a 40-W CO_2_ laser emitting focused laser energy through a small-sized handpiece with success in 74 % of cases with very low pain and complication rate. Sedlmaier et al. [10] reported positive results in 183 children operated with 812 nm 13 W infrared laser diode on an outpatient basis. No bleeding after the procedure was observed. Reichel et al. [9] compared 64 children with recurrent tonsillitis, who had undergone total blunt dissection tonsillectomy and 49 children with tonsillar hyperplasia without tonsillitis, who had undergone partial tonsillectomy with CO_2_ laser. This study has shown that tonsillotomy with CO_2_ laser technique is an effective surgical procedure with a long-lasting effect in patients with tonsillar hyperplasia. The benefits over conventional tonsillectomy are a lower risk for postoperative haemorrhage, reduced postoperative morbidity and accelerated recovery. Therefore, the procedure should be recommended for infected tonsils and might be an option in some patients with mild symptoms of recurrent tonsillitis. Huber et al. [4] described the use of argon laser needle delivery in tonsil hyperplasia surgical treatment in 15 children in comparison with historical CO_2_ laser approach. No haemorrhage was reported, oral food intake and swallowing were normal and postoperative pain was low especially in the third postoperative day favouring argon laser treatment.

Following tonsillectomy, immediate hospital readmission is mandatory in case of secondary bleeding, and an intensive care unit should intensively take care of the patient and, potentially, radiological embolization of the vascular bleeding network might be a lifesaving choice [11]. Positive results have also been previously reported by Ericsson et al. [3] who compared the effects of partial tonsil resection using a radiofrequency technique in 92 children with total tonsillectomy. A follow-up after 1 and 3 years showed that removing only the protruding parts of the tonsils produces the same beneficial long-term effect on recurrent throat infections, if compared to total tonsillectomy. A further study compared the long-term effects of intracapsular partial tonsillectomy using CO_2_ laser technique and traditional blunt dissection tonsillectomy in 41 children aged between 9 and 15 (*n* (CO_2_ laser) = 21); *n* (traditional blunt dissection tonsillectomy) = 20) [6]. Six years after the procedure, they were asked to answer ten questions about the results of the procedure. The findings from this study showed that partial tonsillectomy represents a reliable method with the same positive long-term effects of traditional treatment.

## Aim

The aim of this article was to describe a new strategy using a Doppler-guided fibre optic neodymium-yttrium–aluminium–garnet (YAG) laser to remove up to 80 % of tonsillar tissue as assessed in the long-term postoperative clinical evaluation of the volume of the tonsils at the follow-up, and leaving the capsule in place avoiding any haemorrhagic complication and pain.

## Materials and methods

**Fig. 1:**
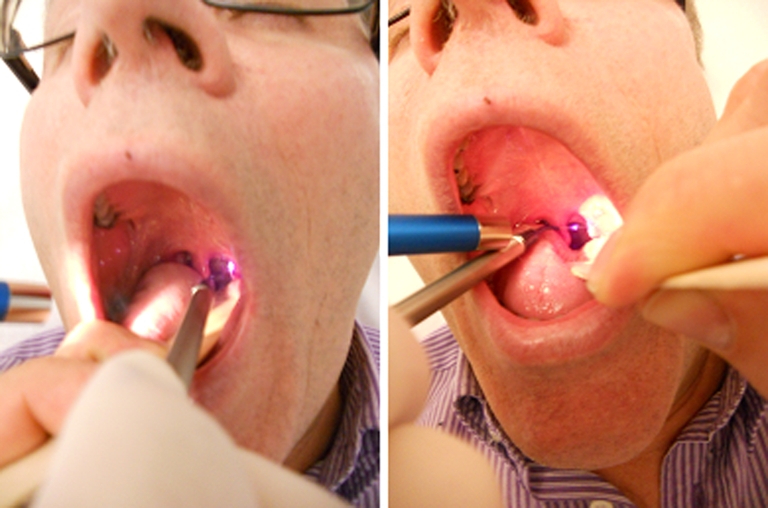
Patient undergoing laser tonsillectomy

**Fig. 2:**
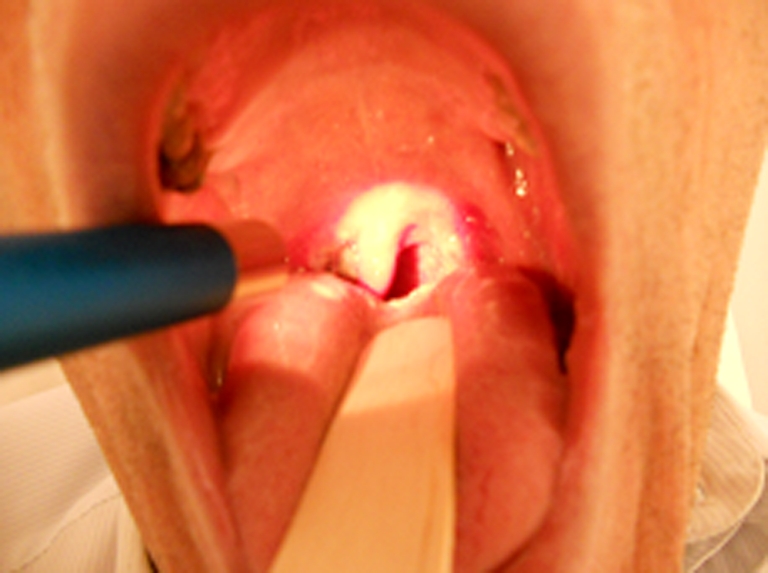
Patient’s examination the day after laser tonsillectomy

**Fig. 3:**
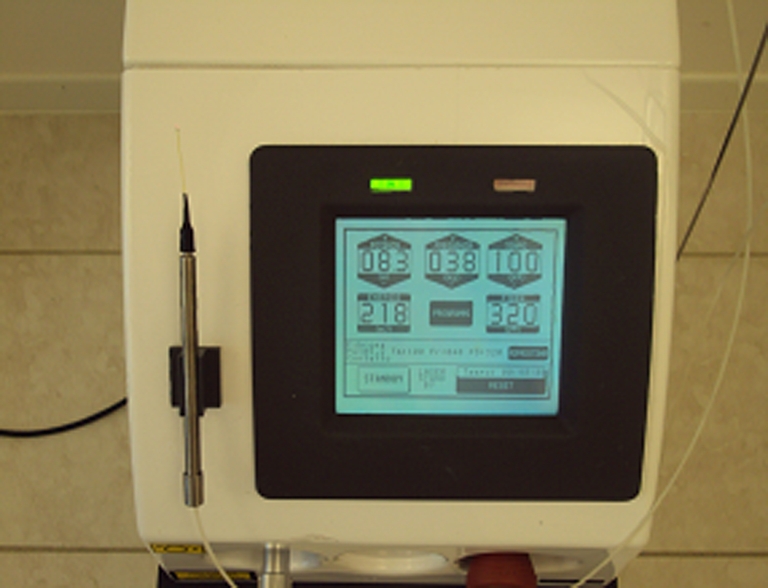
Laser device (Elettronica Valseriana, Bergamo, Italy)

A total of 20 patients (men, *n* = 13; women, *n* = 7), aged between 6 and 63 (35.6 ± 3.4; mean ± Standard Error of the Mean (SEM)), were recruited to participate in the study and signed the informed consent. They were affected by chronic hypertrophic tonsillitis with irradiation to the region of the pharynx, without pus or membranous exudate and recurrent fever. The patients included in this study required a low-invasive surgical procedure to overcome chronic recurrent tonsillar infections. Exclusion criteria for the current study were: cardiomyopathy, renal insufficiency, respiratory insufficiency and pregnancy. The present study was performed at the “Poliambulatorio del Secondo Parere” (Modena, Italy) according to the Helsinki Declaration, and local Internal Review Board (IRB) approval was obtained. The 6-year-old patient was given 0.2 mg/kg Valium (Roche, Milan, Italy) intranasally before the beginning of the procedure. The inferior pole of the tonsil was addressed lowering the tongue and addressing the Doppler-equipped fibre optic tip starting from cephalad (Figs. 1 and 2). The patients with hyperactive gag reflexes were addressed with xylocaine spray over the pharynx, plus pretreatment with intramuscular metoclopramide and domperidone. Each patient, comfortably sitting, was injected with 1 ml of 1 % xylocaine with a 28-gauge needle in each tonsil capsule. Before and after the anaesthetic injection, the vascularization of each tonsil was defined by means of a small-sized Doppler probe 404 (Perimed Instruments, Milan, Italy) coupled along the handle tip of the laser and advanced along the axis of the fibre optic before laser beam delivery. This allowed to identify and carefully recognize the vascular network and avoid any major vascular trunk and/or anomaly during the tonsil burning. A fibre 320 µm in diameter and connected with a 1064 neodymium:YAG (Elettronica Valseriana, Bergamo, Italy; Fig. 3) continuous wave laser was used. The laser fluence was 200–400 J/cm^2^, and the energy output was 10 W. Exposure time was 2–4 s (during channel drill). A minute after the local anaesthesia injection, we started transfixing the capsule with the fibre optic moving up and down along the lymphatic tissue and gradually emptying the tonsil parenchyma by cautious laser heating. We carefully avoided any damage to the previously identified vascular network because the back and forth fibre movement was strictly performed on the trajectory previously explored by the Doppler probe. The procedure required no more than 5–10 min and was intermittently performed on each side, allowing thermal relaxation of the laser-injured tissue. A total of 20 to 50 laser channels were drilled and their mouths rinsed with iced refreshment; a liquid disinfectant was administered at the end of the operation. Adverse events, including dysphagia, fever and cough were evaluated. The patients were discharged from operative chair 10 min after the end of the procedure, with the multi-drilled tonsil surface being carefully inspected. Follow-up examinations were performed at different time points up to 36 months later according to the individual patient’s needs. During the first examination, the general procedure compliance and postoperative pain level were recorded by means of Scott–Huskisson visual analogue scale (VAS). In the long run, the number of relapsing throat inflammation episodes was ascertained, and a clinical examination of the tonsils and pharynx was performed to update the impact of the laser procedure. The protocol enclosed a single session followed by further sessions, if required in case of relapse or inflammation. At the follow-up, the patients were divided into two groups (Table 1), i.e. “complete recovery” if their symptoms had completely disappeared or “improved symptoms” when an improvement was observed in their clinical situation after the procedure (Table 1). The amount of tonsillar tissue removed was measured by means of application of a silicon imprint attached to a stainless steel cylinder.

**Table 1:** Summary of study results

Patient number	Age	Diagnosis	Sessions^*^	Adverse events	Postoperative pain (VAS, mm) measured in the 7 days following the first session (medication used)	Follow-up^**^
1	24	Chronic tonsillitis	1	/	20	24 months/Improved symptoms
2	57	Hypertrophic tonsillitis	2	Moderate sore throat	10	18 months/Complete recovery
3	34	Recurrent tonsillitis	3	Moderate burning and local swelling persisting for 24 h	20	36 months/Complete recovery
4	37	Chronic pharyngotonsillitis	3	/	25	16 months/Improved symptoms
5	26	Relapsing acute tonsillitis	1	/	5	21 months/Complete recovery
6	27	Tonsil remnants with infections	2	/	15	24 months/Complete recovery
7	24	Tonsillar hypertrophy and infections	1	Moderate oedema	20	16 months/Complete recovery
8	56	Recurrent tonsillitis	1	Moderate burning persisting for 48 h	40 (Diclofenac 100 mg capsules [2 times/day for 3 days; IBSA, Lugano, Switzerland])	21 months/Complete recovery
9	21	Relapsing tonsillitis and tonsillar hypertrophy	1	Moderate exudate	30 (Naprosyn 750 mg capsules, [2 times/day for 3 days; Recordati, Italy])	20 months/Complete recovery
10	6	Tonsillar hypertrophy	2	Moderate pain and dysphagia	20 (ice)	24 months /Complete recovery
11	32	Pyogenic tonsil remnants	2	Pain and scanty blood spots	40 (Tranex, 500 mg/5 ml ampoules [2 times/day for 1 day; Malesci Istituto Farmacobiologico, Bagno a Ripoli, Italy], Contramal 100 mg capsules [1/day for a day; Formenti, Milan, Italy])	20 months/Complete recovery
12	51	Tonsillar hypertrophy and relapsing tonsillitis	1	Burning and pruritus	20 (ice)	18 months/ Complete recovery
13	19	Relapsing tonsillitis and laryngitis	2	Moderate discomfort and dryness	15 (Ephynal 300 mg capsules [3 times/day for 2 weeks; Abiogen Pharma, Pisa, Italy] and ice)	15 months/Improved symptoms
14	63	Post-viral tonsillar hypertrophy	1	/	5	19 months/ Improved symptoms
15	34	Chronic tonsillitis	1	Moderate discomfort and cough	20 (ice)	12 months/Improved symptoms
16	44	Tonsillitis and tonsillar hypertrophy	2	/	30 (Toradol 30 mg/ml ampoules [1/day for 2 days; Rome, Italy])	18 months/Complete recovery
17	43	Chronic fibrinous tonsillitis	1	Some transient dysphagia	25 (ice)	14 months/Improved symptoms but one replapsing episode was observed
18	59	Acute relapsing tonsillitis	1	Sore throat	10	18 months/Complete recovery
19	34	Tonsillar hypertrophy and rheumatic fever	1	Slight oedema and swallowing burns	25 (local Xentafid 0.13 % [3 times/day for 5 days; Fidia Farmaceutici, Abano Terme, Italy] and ice)	24 months/Complete recovery
20	21	Waldeyer’s hypertrophy and tonsillar culture positive for Streptococcus viridians	2	Inflammation of the pharynx	35 (Augmentin 375 mg tablets [2 times/day for 3 days; GlaxoSmithKline, Verona, Italy])	24 months/Improved symptoms but one replapsing episode was observed

^*^Number of sessions varied according to the individual patient’s clinical situation

^**^Follow-up varied according to the individual patient’s clinical situation

## Results

A maximum of three sessions was necessary (mean ± SEM; 1.55 ± 0.15). Among the 20 patients who participated in the present study, no major untoward effect, including haemorrhage-related complications, was observed. The vascular network preliminary exploration with the Doppler probe, immediately followed by the laser tip advancement along the vascular tonsil axis, took very few minutes. Treatment was well tolerated even in patients displaying a low pain threshold, and no dropout or uncompleted procedure was noted. The back and forth fibre optic movements across the lymphatic tissue and far enough from the vascular network created multiple empty channels (between 30 and 120) without completely collapsing the structure. Minor complications are summarized in Table 1. General discomforts, such as cough, pruritus, dysphagia and dryness, were also common among the patients (Table 1). The summary of postoperative pain in the 7 days following the first session, as measured by Scott–Huskisson VAS, was 21.5 ± 2.2 mm (mean ± SEM; range 5–40 mm) and easily counteracted by means of ice packages applied around the neck, nonsteroidal anti-inflammatory drugs and other drugs according to the individual patient’s need (Table 1). During the 12–36-month follow-up patients showed improved symptoms (*n* = 7) and complete recovery (*n* = 13). A relapse episode was observed in two patients. The procedure was safe since no serious adverse events, including haemorrhagic complications, occurred. The pain perceived was of low intensity, and after the procedure, the patient was allowed to drink and eat without any further complications.

## Conclusions

The present study describes the use of a Doppler-guided fibre optic neodymium-YAG laser to remove up to 80 % of tonsillar tissue. We named this procedure “cribriform intracapsular tonsillectomy” or Swiss-cheese laser tonsillectomy. This procedure consists in a multi-drilled total or subtotal parenchyma removal, leaving the membrane and vascular stromal network in place. At the 12–36 month follow-up, among 20 patients, 7 patients showed an improvement in symptoms and 13 showed a full recovery. Two patients reported a relapse episode. Treatment was well tolerated with no untoward effects. Postoperative pain at day 7 following the first session was between 5 and 40 mm. Therefore, the present approach allows achieving relatively painless removal of tonsillar tissue safely and with promising results.

According to the literature, several tonsillectomy-related complications may occur. Selected tonsillar tissue destruction by a small-sized laser fibre optic is a safer option compared with the traditional cold knife approach or electrosurgery. In this article, we describe a procedure that encloses a Doppler-assisted neodymium-YAG surgical procedure to identify vascular topography, thus preventing any damage to the vascular network during the fibre optic insertion and, through the capsule, into the affected parenchyma.

We argue that cold knife tonsillectomy is cumbersome and time-consuming, and we suggest to preliminarily explore, step by step, by means of Doppler probe, the tonsillar parenchyma along the fibre optic direction. The procedure is followed by laser parenchyma ablation. In our experience, the creation of multiple channels into the lymphatic tissue was very effective to remove either hyperplastic or chronically infected tonsils, and offered a better quality of life in terms of pain control, immediate swallowing, eating and precocious recovery. As to the laser source, we had preliminary comparative experience with argon, CO_2_, fibre optic diode and neodymium-YAG laser. Argon and CO_2_ laser did not reach any substantial advancement in the surgical outcome and were quite cumbersome to be used across the tonsils resulting in relevant pain. The fibre optic innovation opens new perspectives in tonsillar surgery, rendering the procedure very easy and individually tailored to the tonsils’ anatomy and pathology. Our strategy, consisting in coupling Doppler and fibre optic in the same handle, was fundamental to achieve the goal of the procedure without risks. The neodymium-YAG source is the most effective in terms of simple use and energy quality as it is an optimal choice in the operating room. We routinely adopted the 320-μm fibre optic diameter, being the safest possible in terms of minimal damage to the surrounding tissue and the selectivity of minor vessel coagulation. In fact, no haemorrhage was observed in our cases, and haemostasis was excellent and safe due to the previous identification of the major tonsillar vessels and, more generally, to the vascular parenchymal network. This laser approach, compared with cold knife tonsillectomy, leaves some sterilized necrotic debris and cluster particles that might elicit a better immunological response against infectious agents. It can also explain the long-term successful outcome of the fibre optic procedure extended to the pharyngo-tonsillar area that is very often involved in the inflammatory process. In conclusion, fibre optic laser procedure can be the gold standard for tonsil surgery in the third millennium for safety reasons, acceptable cost–benefit ratio, precise targeting of the beam across affected tissues and short- and long-term recovery.
